# The management of uterine tumor resembling an ovarian sex cord tumor (UTROSCT): case series and literature review

**DOI:** 10.1186/s12957-024-03319-3

**Published:** 2024-02-03

**Authors:** Jie Lin, Linying Liu, Linghua Wang, Ning Ma, Kailin Zhang, Ning Xie, Haijuan Yu, Sufang Deng, Yang Sun

**Affiliations:** 1 Department of Gynecology, Clinical Oncology School of Fujian Medical University, Fujian Cancer Hospital, Fuzhou, Fujian Province China; 2 Department of Radiology, Clinical Oncology School of Fujian Medical University, Fujian Cancer Hospital, Fuzhou, Fujian Province China; 3 Department of Pathology, Fujian Medical University Union Hospital, Fuzhou, China

**Keywords:** UTROSCT, Prognosis, Hysterectomy, Conservative management

## Abstract

**Aims:**

To present a case series of 11 rare uterine tumors resembling ovarian sex cord tumors (UTROSCTs), and review the literature on this topic to offer up-to-date treatment management for UTROSCTs.

**Method:**

Eight cases from Fujian Cancer Hospital between January 2017 and May 2023 and three patients from Fujian Union Hospital between October 2012 and October 2020 were retrospectively reviewed. All cases were pathologically confirmed as UTROSCTs by two senior and experienced pathologists. Clinical behaviors, medical data, histopathological features, therapy approaches, and survival outcomes were discussed.

**Results:**

The median age at initial diagnosis was 53 years (29–70 years). 3 (27.3%) patients were under 40. Seven cases presented with abnormal vaginal bleeding, one with menstrual disorder, one with abnormal vaginal secretion, and two patients were accidentally found by physical examination without any symptoms. Three patients were initially misdiagnosed with endometrial cancer by MRI. Curettage was performed in all cases. Nine of them were well diagnosed by routine curettage, except for two samples, which were identified after surgery. Immunohistochemical biomarkers, such as CD99, Desmin, WT-1, CK, Vimentin, SMA, α-Inhibin, Ki67, CD56, ER, PR, and CR, tend to be positive in UTRO SCs patients. Six patients underwent hysterectomy with bilateral salpingo-oophorectomy. Two cases received a radical hysterectomy with bilateral salpingo-oophorectomy, retroperitoneal lymph node dissection, and omentum dissection. Three UTROSCTs were under observation after mass resection. The median PFS was 24 months (range 1–125 months).

**Conclusion:**

UTROSCT is a rare mesenchymal tumor with low malignant potential. Treatment modalities should be carefully considered to balance the therapy outcomes and patient needs. Surgery conservative management might be suitable for young women with fertility desires.

## Introduction

Uterine tumor resembling ovarian sex cord tumor (UTROSCT) is a sporadic and controversial disease with unclear origins, named for resembling the morphology of ovarian sex cord tumors [1]. It mainly occurs in women in their 50 s and occasionally in young women with childbearing needs [2]. UTROSCT patients always present with irregular vaginal bleeding or chronic pelvic pain. However, some can be discovered by accidents [3]. Although UTROSCT is generally considered benign or has a low-malignant potential, it could metastasize sometimes. There are no established treatment protocols for UTROSCT so far. Surgical intervention is usually recommended with a favorable prognosis.

Although there has been modest literature on UTROSCT in recent years, focusing mainly on clinicopathological characteristics and gene variations, relatively few advances have been made in treatment strategies due to the low incidence. Here we reported 11 UTROSCT cases with different therapy approaches together with an updated literature review to expand our knowledge of UTROSCT management.

## Materials and methods

### Clinical data

A total of 11 UTROSCT patients from two institutions from October 2012 and October 2020 were included. Intrauterine tissue and surgical specimens were reviewed and confirmed by two senior and experienced pathologists. The clinical and follow-up information was obtained through medical files and telephone contact. We collected information such as menopausal status, parity, symptoms, tumor positions, tumor biomarkers, image data, immunohistochemical biomarkers, treatment approaches, and survival status. May 31, 2023, was the final censoring date for assessing the survival time.

### Interventions

All patients underwent diagnostic curettage, three of which were hysteroscopically assisted. For further steps, six patients underwent hysterectomy with bilateral salpingo-oophorectomy, two cases received radical hysterectomy with bilateral salpingo-oophorectomy, retroperitoneal lymph node dissection, and omentum dissection, and three were under observation after tumorectomy due to their desires for children-bearing in the near future. All surgeries were performed by experienced gynecology surgeons. After treatment, patients were advised to have follow-up visits every three months in the first 2 years, every six months in the 3–5 years, and once a year after 5 years.

## Results

### Clinical features and treatment outcomes

The age ranged from 29 to 70 (median = 53) at the initial diagnosis. Seven cases presented with vaginal bleeding, one with menstrual disorder, and one with vaginal secretion. While two showed no symptoms. No elevation of serum tumor biomarkers such as CA125, CA199, and CEA were observed in 11 cases. The mass positions were in the endometrium (4 cases) and myometrium (7 cases). None of the patients received further postoperative treatment. The median follow-up time was 24 months (1–125 months), and no recurrence or metastasis was noted. Details are presented in Table 1.

**Table 1:** Clinical features of UTROSCT patients

Case	Age	Menopausal status	Parity	Symptoms	Tumor position	Tumor biomarkers			Treatment approaches	Recurrence
						CA199 U/ml	CEA ng/ml	CA125 U/ml		
1	39	No	2–0-0–2	Vaginal bleeding	Submucosa (myometrium)	13.61	2.59	9.06	Mass resection	No (1 month)
2	34	No	1–0-0–1	Menstrual disorder	Submucosa (myometrium)	−	NA	NA	Mass resection	No (2 months)
3	29	No	1–0-1–1	No symptoms	Submucosa (myometrium)	−	NA	NA	Mass resection	No (54 months)
4	40	No	0–0-0–0	No symptoms	Endometrium	9.39	NA	23	Hysterectomy	No (24 months)
5	67	Yes	2–0-0–2	Vaginal bleeding	Submucosa (myometrium)	17.72	2.5	6.64	Hysterectomy	No (5 months)
6	53	No	2–0-0–2	Vaginal bleeding	Endometrium	6.64	1.49	12.44	Hysterectomy	No (6 months)
7	55	Yes	2–0-1–2	Vaginal bleeding	Submucosa (myometrium)	NA	NA	NA	Hysterectomy	No (66 months)
8	70	Yes	5–0-1–5	Vaginal bleeding	Submucosa (myometrium)	NA	NA	NA	Hysterectomy	No (125 months)
9	52	Yes	2–0-0–2	Vaginal bleeding	Endometrium	14.64	2	8	Hysterectomy	No (29 months)
10	57	Yes	3–0-0–3	Vaginal secretion	Endometrium	NA	NA	NA	Radical surgery	No (31 months)
11	59	Yes	2–0-0–2	Vaginal bleeding	Submucosa (myometrium)	**-**	1.54	NA	Radical surgery	No (13 months)

*Abbreviations*: *NA* not available, − negative

## Radiological imaging characteristics

**Fig. 1:**
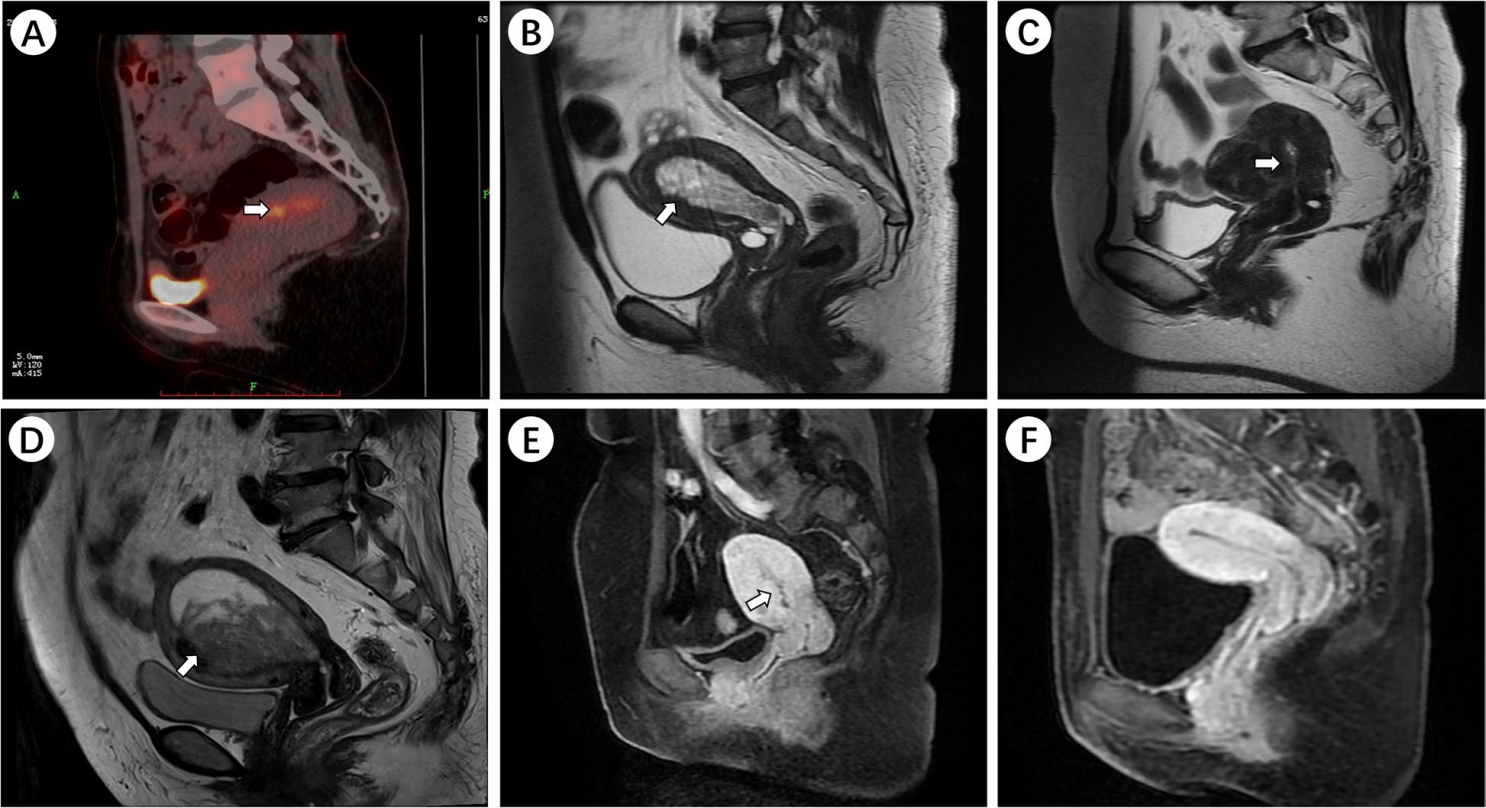
UTROSCT (arrow) in different images of PET-CT and MRI. **A** A max SUV was 4.5 in the myometrium. **B**–**D** UTROSCT is mildly hypointense compared to endometrium signals on sagittal T2 sequence. **E** UTROSCT is similar to uterus signal and well-delineated on dynamic contrast-enhanced sequence. **F** No mass observed after curettage

Radiological imaging sometimes could have been deceiving due to the small population size and limited knowledge about UTROSCT. The max SUV value in the myometrium is 4.5 under PET-CT examination after hysteroscopic curettage in case 3 (Fig. 1A) with conservative therapy. An increasing endometrial thickness was observed in cases 4 and 10 (Fig. 1B, C). A mass with myometrial invasion of case 11 which was misdiagnosed as endometrial cancer was finally proved to be UTROSCT (Fig. 1D). Case 6 was also suspected as endometrial cancer by MRI examination (Fig. 1E), so as case 10. No residual tumor was noted after curettage (Fig. 1F). Other patients didn't conduct a radiology examination. The radiological imaging findings are presented in Fig. 1.

### Pathological findings

**Fig. 2:**
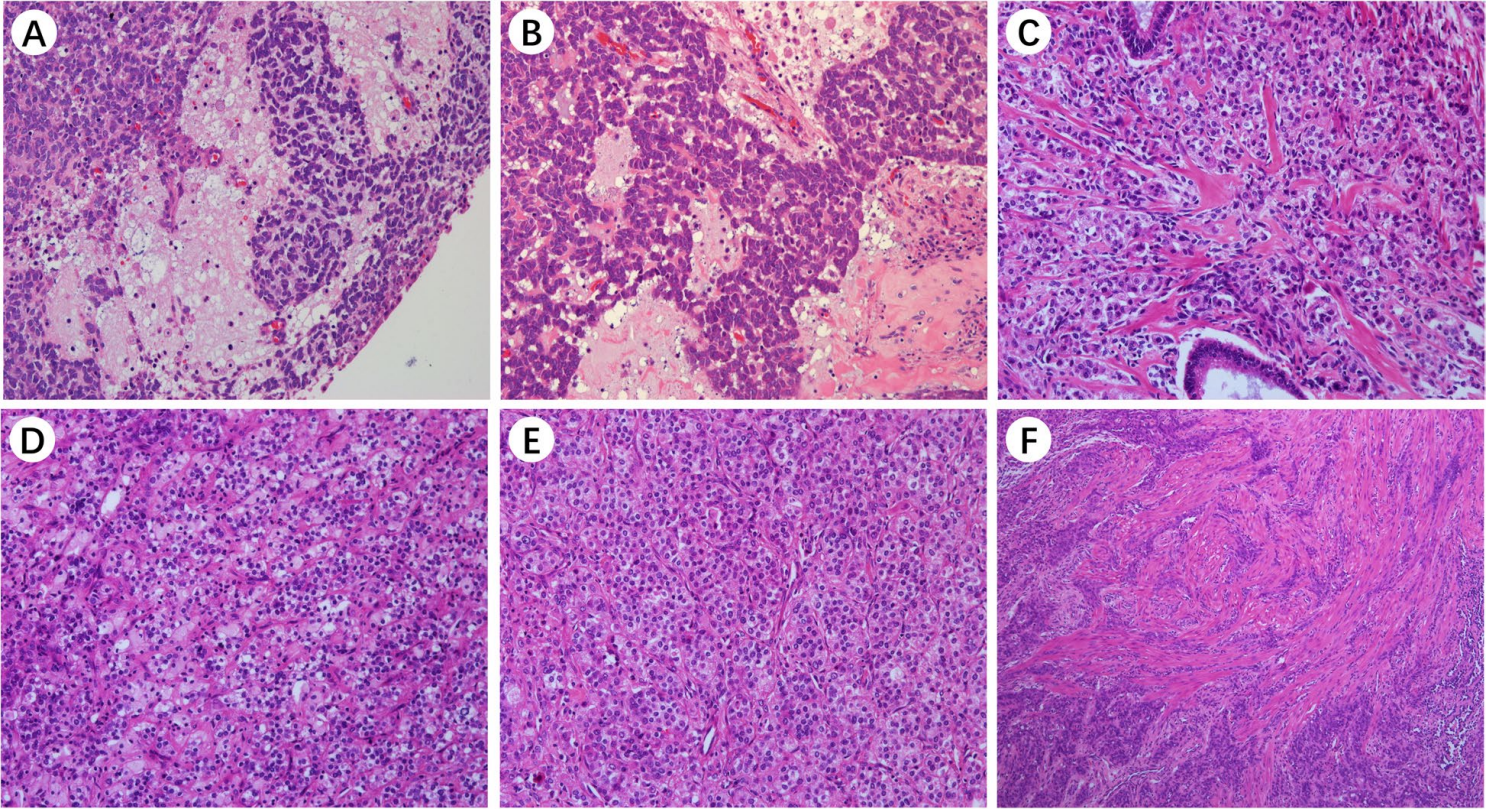
Morpholohistopathology of UTROSCT. UTROSCT is characterized by various architectures including **A** myxedema, **B** sex cord-like structure, **C** collagen degeneration, **D** foam-like cell, **E** organ-like structure. **F** muscle layer infiltration (H&E × 10)

Glandular epithelium, basal-like, and myoepithelial cells are the three main kinds of cells that could be found in UTROSCT patients. Under the light microscope, we could see specific common structures: myxedema, sex cord-like structure, collagen degeneration, foam-like cells, organ-like structure, and muscle layer infiltration, which are often mixed and concurrence (Fig. 2).

**Fig. 3:**
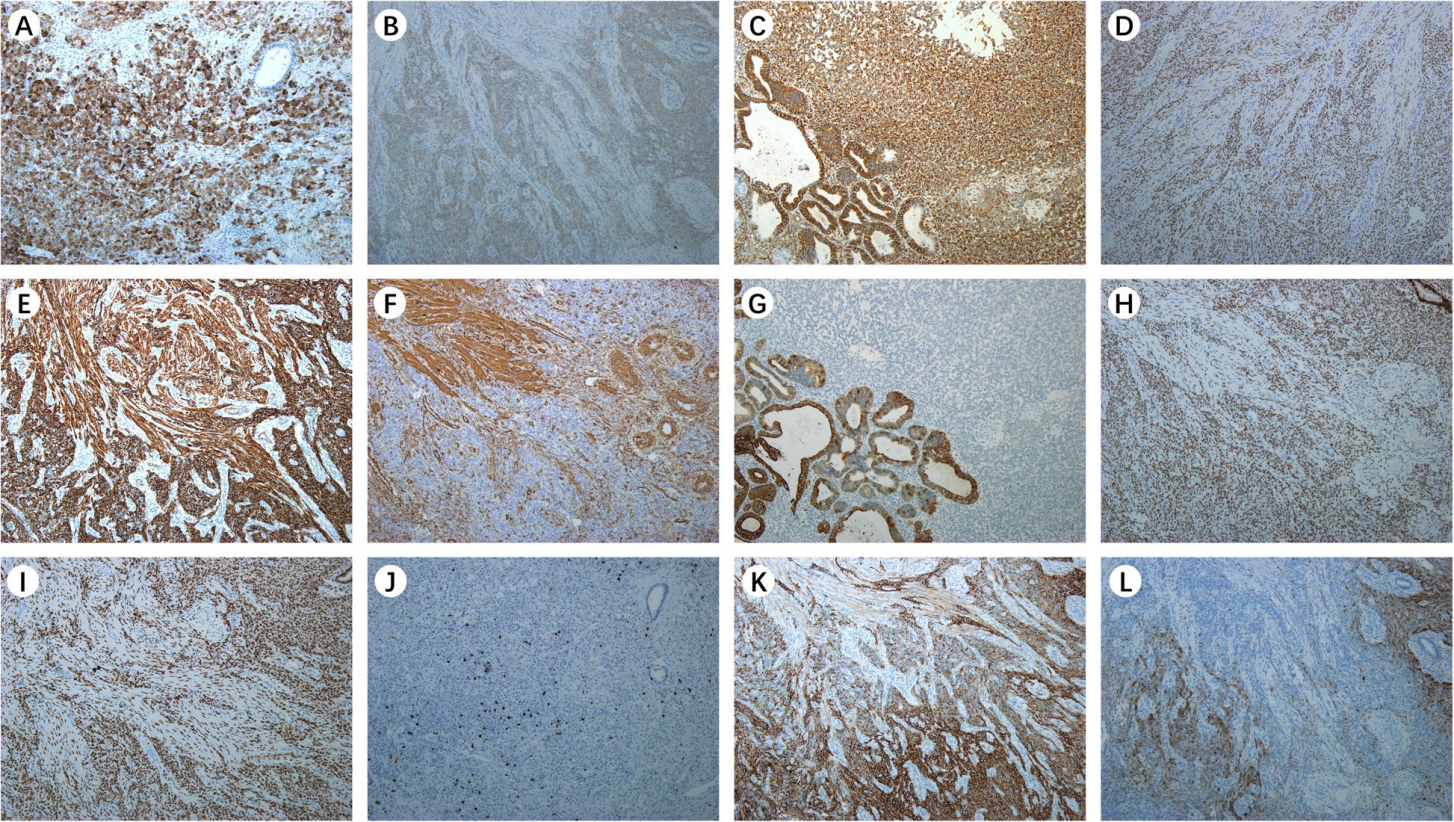
Immunohistochemical biomarkers of UTROSCT. **A** α-inhibin. **B** CD99. **C** Vimentin. **D** WT-1. **E** desmin. **F** SMA. **G** CK. **H** ER. **I** PR.** J** Ki67. **K** CD56. **L** Calretinin (IHC × 10)

Pathology is the golden standard for UTROSCT diagnosis. Patients with UTROSCT showed a diverse phenotype profile, according to immunohistochemistry performed on UTROSCT patients (Fig. 3, Table 2). Biomarkers such as inhibin, CD99, CD56, WT-1, and CK tend to be positive in UTROSCTs. Most tumors had exhibited at least one of the standard sex-cord markers, including CD99, WT-1, and α-Inhibin (Table 2). The positive expression rates of α-inhibin (Fig. 3A), CD99 (Fig. 3B), Vimentin (Fig. 3C) and WT-1 (Fig. 3D) were 3/10, 7/9, 5/6, 8/8, respectively. Almost all patients (9/11) expressed one or more smooth muscle markers, and the positive expression rates of desmin (Fig. 3E) and SMA (Fig. 3F) were 8/10 and 6/10, respectively. CK (Fig. 3G) as an epithelial biomarker was positive in most cases (10/11), and hormone receptors like ER (Fig. 3H) and PR (Fig. 3I) expressed variably among UTROSCTs. A low Ki67(Fig. 3J) index was observed among the patients (median 5%, range 2–30%). While CD56 (Fig. 3K) and Calretinin (Fig. 3L) are often used for differentiated diagnosis.

**Table 2:** Immunohistochemical biomarkers in the 11-patient cohort

Case	CD99	Desmin	WT-1	CK	Vimentin	SMA	α-Inhibin	Ki67	CD56	ER	PR	CR
1	NA	+	+	+	NA	+	−	1%	+	+	+	+
2	+	+	+	+	+	+	−	2% +	+	+	+	NA
3	+	+	+	+	+	+	+	1% +	NA	NA	NA	+
4	−	−	NA	−	+	−	−	10% +	+	−	−	+
5	−	NA	+	+	NA	+	+	10% +	+	+	+	+
6	+	+	+	+	−	−	+	10% +	NA	+	+	NA
7	+	+	NA	+	NA	+	−	2%	NA	+	+	+
8	+	−	NA	+	+	−	−	NA	NA	NA	NA	NA
9	+	+	+	+	NA	+	−	30% +	+	+	NA	+
10	NA	+	+	+	NA	−	−	40%	+	+	NA	NA
11	+	+	+	+	+	NA	NA	10% +	NA	+	+	−

*Abbreviations*: + positive, − negative, *NA* not available

## Discussion

In 1976, Clement and Scully first reported certain kinds of rare uterine tumors with histologic resemblances to ovarian sex-cord tumors [4]. Those types of tumors could be divided into two groups. The first group is endometrial stromal tumors with sex cord-like elements (ESTSCLE) with a particular propensity to recur or metastasize, and UTROSCT is the other group [5]. UTROSCT is rare and usually recognized as a benign tumor. Clinical observations and literature data indicated a better prognosis [6]. However, sporadic reports showed it could metastasize and recur sometimes. In 2017, Moore documented 34 UTROSCT cases, of which about 23.5% exhibited malignant behavior [7]. Due to the potential malignancy and limited experience, treatment recommendations have not been well established.

Surgery is recommended as the preferred treatment for UTROSCT patients [8]. Hysterectomy or mass resection alone are possible options for its management [9]. A systematic review of the literature on UTROSCTs’ treatment approaches is detailed in Table 3. With advancements in medicine and humanities, individualized-oriented tumor treatments have been deeply rooted in people’s hearts. We should pay more attention to patients with special needs or in different conditions, and then make personalized plans to balance the therapy outcomes and their needs.

**Table 3:** Different therapies and outcomes of UTROSCT patients reported in the literature (2014 ~ 2023)

Present cases	No	Year	Age	Outcome (follow-up)
Hysterectomy ± bilateral salpingo-oophorectomy				
Gutierrez-Pecharroman, et al. [10]	1	2014	49	Ned (18 months)
Bakula-Zalewska, et al. [6]	4	2014	50–63	Ned (3–14.5 years)
Moore, et al. [7]	31	2017	26–86	7/31 recurrence (6–135 months)
Kondo, et al. [11]	1	2018	63	1/1 recurrence (6 years)
Croce, et al. [12]	1	2019	72	1/1 recurrence (17 months)
Dubruc, et al. [13]	1	2019	56	Ned (4 months)
Zhang, et al. [14]	2	2019	33, 64	Ned (12 days–1 year)
Lee, et al. [15]	2	2019	34, 47, 59	Ned (116 months–20 years)
Vilos, et al. [16]	2	2019	47, 52	Ned (1–3 years)
Dimitriadis, et al. [17]	1	2020	46	1/1 recurrence (2 years)
Grither, et al. [18]	1	2020	69	Ned (8 months)
Chang, et al. [19]	1	2020	57	1/1 recurrence (30 months)
Bennett, et al. [20]	3	2020	32, 37, 54	3/3 recurrence (7–32 years)
Kaur, et al. [21]	5	2020	42–50	1/5 recurrence (1 month–2 years)
Goebel, et al. [22]	10	2020	34–74	1/10 recurrence (1–319 months)
Zhou, et al. [8]	1	2021	51	Ned (58 months)
Devereaux, et al. [23]	1	2021	42	1/1 recurrence (6 months)
Shibahara, et al. [24]	1	2022	77	Ned (12 months)
Wang, et al. [3]	1	2022	42	Ned (2 months)
Pang, et al. [25]	2	2022	42, 46	Ned (4–35 months)
Ye, et al. [2]	4	2022	40–70	Ned (uncertain)
Zhou, et al. [26]	1	2023	49	Ned (1 months)
Lu, et al. [27]	11	2023	37–60	Ned (11–141 months)
Radical surgery				
Umeda, et al. [28]	2	2014	38, 57	Ned (11 months–8 years)
Mačák, et al. [29]	1	2015	53	Ned (10 months)
Cetinkaya, et al. [30]	1	2016	52	Ned (17 months)
Kuznicki, et al. [31]	1	2017	49	Death (15 months)
Fan, et al. [32]	1	2018	62	Ned (20 days)
Segala, et al. [33]	1	2019	62	Ned (5 years)
Sato, et al. [34]	1	2020	57	Ned (3 years)
Conservative treatment				
Bakula-Zalewska, et al. [6]	2	2014	24,25	Ned (4.5–7 years)
Watrowski, et al. [35]	1	2015	22	Ned (28 months)
Jeong, et al. [36]	1	2015	32	Ned (47 months)
De Franciscis, et al. [37]	1	2016	38	Ned (60 months)
Moore, et al. [7]	2	2017	12, 36	Ned (27–63 months)
Schraag SM, et al. [38]	2	2017	24, 28, 72	2/2 recurrence (6–39 months)
Lee, et al. [15]	1	2019	34	Ned (49 months)
Goebel, et al. [22]	1	2020	38	Ned (50 months)
Carbone, et al. [39]	2	2021	25, 30	Ned (96 months,16 months)
Ye, et al. [2]	1	2022	40	Ned (uncertain)
Lu, et al. [27]	6	2023	27–50	Ned (17–55 months)

*Abbreviations*: *Ned* no evidence of disease. Conservative treatment: tumorectomy, myomectomy, and electrical resection, etc.

### Hysterectomy for UTROSCTs in the middle and old age

Hysterectomy resection with or without salpingo-oophorectomy should be a primary option for middle-aged or older women with UTROSCT, especially in patients whose follow-up cannot be guaranteed [9]. Baris Boyraz recorded that hysterectomy is the prior surgery approach in 71 out of 75 UTROSCT patients [40]. Besides, most patients had good survival outcomes simply by removing the uterine [21, 22, 40]. Moreover, some scholars claimed recurrence cases previously treated by hysterectomy [11, 12, 20] could still get prolonged survival by completely removing the recurrent mass. In our study, 6 out of 11 patients treated by hysterectomy did not experience recurrence and metastasis after a median follow-up time of 26.5 months (range 5–125 months). However, we still need a longer follow-up time and more cases to make survival outcomes more convincing.

### Radical surgery for UTROSCTs with high risk of recurrence or metastasis

UTROSCT has uncertain malignant potential due to its low recurrence rate [7]. Distant metastasis and local recurrence have been occasionally reported [11, 22, 28, 29]. Our review found that the recurrence rate was low (19/117) during the median follow-up time between 20 days and 32 years. Currently, predictive features of aggressive UTROSCTs are poorly understood. Risk factors, such as myometrial invasion, serosal involvement, lymph-vascular space invasion (LVSI), GREB1-NCOA2 fusion, NCOA2 or NCOA3 [23, 41, 42], and high mitotic activity [40, 43]have been proven to be associated with tumor recurrence. Baris Boyraz concluded that high-risk UTROSCT showed more than three of the following five features: at least moderate cytologic atypia, tumor size > 5 cm, above 3 mitoses/10 at high power fields (HPFs), marginal infiltration, and necrosis [40].

High-risk UTROSCT patients could benefit from radical surgery. Miho believed hysterectomy alone was associated with a higher rate of recurrence. Extended radical surgery, including bilateral salpingo-oophorectomy, lymphadenectomy, and omentectomy, may reduce the recurrence rate of UTROSCT with sarcomatous features [34]. Based on the review in Table 3, no recurrence or distant metastasis was observed among 8 UTROSCT patients treated with radical surgery, indicating radical surgery seemed to be the optimal choice for high-risk patients. However, it should be attention that high-risk UTROSCT is often detected incidentally after a hysterectomy surgery, which makes the initial treatment insufficient. For this reason, the roles of adjuvant therapies should be carefully considered. Surprisingly, the literature showed adjuvant treatments such as chemotherapy, hormone therapy, or radiotherapy were still in debate for the UTROSCTs with high risks of recurrence or metastasis [35]. Oriana Marrucci reported a rare vaginal vault recurrence case 5 years after total hysterectomy with bilateral salpingo-oophorectomy. She had a favorable prognosis with only a second surgery [44]. Shigeaki Umeda also represented a case with epiploic appendix metastasis, who was treated with surgery and has been followed up for 8 years without evidence of recurrence [28]. However, Michelle presented an unusual case of an aggressive UTROSCT who underwent neoadjuvant chemotherapy followed by optimal cytoreductive surgery and adjuvant chemotherapy and still died 15 months after her initial diagnosis [31]. Therefore, the selection of auxiliary treatments needs to consider the specific situations of UTROSCT patients.

Still, the role of hormone therapy is also controversial. Sabrina M. Schraag recorded that surgery and endocrine treatment were applied for a young UTROSCT with recurrence. She was disease-free 34 months after the last surgery and was still on a monitor [38]. High-dose progesterone therapy was also applied after surgery in a UTROSCT with pelvic lymph node metastasis, and no recurrence was observed [28]. While Daisuke Endo pointed out that hormone therapy may be ineffective for recurrent tumors [45]. Some scholars even believed hormone therapy such as tamoxifen progressed this disease [33]. In our study, none patients received additional treatments, and no recurrences were observed. To be pointed out, two cases received radical surgery due to malignancy features shown in MRI images and pathology ambiguity. Based on the above, we believe the choice of surgical approaches should be carefully considered based on whether she is a high-risk UTROSCT.

### Conservative therapy for UTROSCTs with fertility desire

With the development of minimally invasive surgery, tumorectomy by hysteroscopy or laparoscopy could be expected in young UTROSCTs who desire to preserve their organs and fertility. Many scholars reported successful pregnancies in young UTROSCT women treated with fertility-preserving surgery and had no sign of recurrence [38, 39], even in a myometrial invasion case [36]. Only one relapsed case after 20 months of conservative surgery [38]. In our review, all UTROSCTs with conservative therapy had favorable prognoses. Most researchers believed UTROSCT patients could maintain fertility without affecting the survival rate. In our study, three cases (Nos. 1, 2, and 3) were done with mass resection. After a comprehensive examination without signs of residue tumor, case 2 opted for surveillance. The menstruation returned to normal after conservative therapy. She has been followed up for two months with disease-free. In case 3, a PET/CT showed a max SUV value of 4.5 in the myometrium, which cannot be confirmed as postoperative changes or residue tumors. After exhaustive discussions with patients, she still chose to observe due to her eager solid of fertility preservation. Surprisingly, there are no signs of recurrence or metastasis after a follow-up of 54 months. Case 1 had a myomectomy and showed no sign of recurrence. She strongly desired to preserve her organs and decided to be closely observed. A conservative, uterus-preserving treatment appears justified in those whose close follow-up can be guaranteed, even in high-risk ones. Once recurrence, patients could still get a favorable prognosis after salvage surgery [7]. Further investigations are needed to prove the safety of organ-preserving strategy in UTROSCT.

UTROSCT is somehow inert and quickly got the attention of irregular uterine bleeding. All patients in our research were diagnosed in the early stages. A hysterectomy seems enough. However, a 40-year-old female still firmly chose to have extra salpingo-oophorectomy due to psychological fear of tumor recurrence, even after being fully educated on UTROSCT. Moreover, pre-surgery examinations, such as MRI images and curettage pathology, could sometimes be confusing, leading to overtreatment for oncological safety. Clinicians should focus more on aggressive UTROSCT features patients shared, such as tumor size, myometrial invasion, LVSI, and gene fusion, to determine the surgical approaches. Working with an experienced pathologist is necessary.

As far as we know, few articles have summarized and compared the different therapy approaches to UTROSCT. Our study gave a comprehensive knowledge of UTROSCT therapy methods. However, the main limitation of our study is that it was designed retrospectively. Specific information about related cases is lacking, the follow-up time for the young UTROSCT needs longer, and genetic alterations and molecular detection remain to be explored. However, we still contributed 11 UTROSCTs to the medical literature, which might aid clinical decision-making.

## Conclusion

UTROSCT patients are often diagnosed in the early stage. Hysterectomy with or without salpingo-oophorectomy should be the primary treatment for UTROSCT patients. Radical surgery is a favored choice for patients with invasive features. Mass resection is safe for young fertility women in clinical surveillance. Further studies are needed to validate these findings.

## Data Availability

The data used or analyzed in the current study are available from the corresponding author upon reasonable request.
